# Invariance Principle for Lifts of Geodesic Random Walks

**DOI:** 10.1007/s10959-026-01480-x

**Published:** 2026-02-23

**Authors:** Jonathan Junné, Frank Redig, Rik Versendaal

**Affiliations:** Delft Institute of Applied Mathematics, TU Delft, Mekelweg 4, 2628 CD Delft, Netherlands

**Keywords:** Invariance principle, Geodesic random walks, Horizontal Laplacian, Riemannian Brownian motion, Riemannian submersion

## Abstract

We consider a certain class of Riemannian submersions $\pi : N \rightarrow M$ and study lifted geodesic random walks from the base manifold *M* to the total manifold *N*. Under appropriate conditions on the distribution of the speed of the geodesic random walks, we prove an invariance principle, i.e., convergence to horizontal Brownian motion for the lifted walks. This gives us a natural probabilistic proof of the geometric identity relating the horizontal Laplacian $\Delta _{\mathcal {H}}$ on *N* and the Laplace–Beltrami operator $\Delta _M$ on *M*. In the setting where *N* is the orthonormal frame bundle *O*(*M*), this identity is central in the Malliavin–Eells–Elworthy construction of Riemannian Brownian motion.

## Introduction

In this paper, we consider geodesic random walks on a Riemannian manifold (*M*, *g*) and their horizontal lift into a manifold $(N,\tilde{g})$ such that there is a Riemannian submersion $\pi : N\rightarrow M$. A motivating example of this setting is the orthonormal frame bundle $\pi _{O(M)}: O(M) \rightarrow M$ of a Riemannian manifold. This example is the basis of the Malliavin–Eells–Elworthy construction of Brownian motion. The important point in this setting is that the horizontal Brownian motion has as a generator the horizontal Laplacian which is a sum of squares of globally defined vector fields; i.e., it is in *Hörmander form*

$$\begin{aligned} \Delta _{\mathcal {H}}= \sum _{i=1}^d H_i^2, \end{aligned}$$

where *d* is the dimension of the manifold. Because of this, the Markov process $\{U(t)\}_{t\ge 0}$ generated by $\Delta _{\mathcal {H}}$ can be constructed as the solution of a Stratonovich SDE driven by an $\mathbb {R}^d$-valued Brownian motion $\{W(t)\}_{t\ge 0}$, namely (see the monograph of Hsu [1]),

$$ dU(t)=\sum _{i=1}^d H_i(U(t))\circ dW^i(t). $$

Moreover, the Brownian motion on the manifold is the projection of this horizontal Brownian motion, based on the fact that

1.1 $$\begin{aligned} \Delta _{\mathcal {H}} (f\circ \pi ) = (\Delta _M f) \circ \pi \end{aligned}$$

for all smooth $f:M\rightarrow \mathbb {R}$. The proof of identity (1.1) in [1] is based on an explicit but somewhat involved computation, using the $\pi$-relations between the covariant derivative and its lifted counterpart. Beyond the setting of the orthonormal frame bundle, horizontal Brownian motion is extensively studied in Baudoin’s monograph [2].

Brownian motion on *M* can be obtained as a scaling limit of geodesic random walks as initially considered by Jørgensen [3]. It is therefore natural to lift these walks horizontally in order to obtain horizontal Brownian motion in the scaling limit. As a consequence of such a weak convergence result, the horizontal Brownian motion on the total manifold *N* and the Brownian motion on the base manifold *M* are then $\pi$-related automatically. It is precisely the aim of our paper to prove this result for a class of geodesic random walks, in the setting of Riemannian submersions, which is the framework in the monograph [2]. In several sub-Riemannian settings, motivated by different contexts such as Carnot groups, equivalence of Laplacians, and horizontal sub-Laplacians as limits of Hamiltonian flows, several authors have studied scaling limits of geodesic random walks; see [4–8].

First, in Sect. 2 we introduce the horizontal random walks and recall the notion of horizontal lift. Second, in Sect. 3 we consider general submersions for which we prove the $\pi$-relation as a corollary of an invariance principle, and we provide several examples, including the orthonormal frame bundle, which we discuss in detail.

## Random Walks and Horizontal Random Walks

In this section, we introduce the stochastic processes we study, namely, horizontal random walks. To do so, we first introduce the analog of random walks in *M*, so-called geodesic random walks, following [3, 9]. Afterward, we explain how these geodesic random walks can be lifted to the total space *N* along a Riemannian submersion $\pi :N \rightarrow M$.

### Geodesic Random Walk

We consider a *d*-dimensional geodesically complete Riemannian manifold *M* with metric *g*, and denote by $T_pM$ the tangent space of *M* at $p\in M$. In order to describe increments of our random walks, we have to consider a collection of probability measures $\mu _p$ on $T_p M$ called a distribution of *increments*; the nomenclature being inspired from [3] where $\mu _p$ describes the direction in which the random walk follows a geodesic when starting from *p*.

More precisely, we define the following Markov processes based on $\{\mu _p\}_{p \in M}$:

#### Definition 2.1

1. The *discrete-time unit speed random walk* $\{S_k\}_{ k\in \mathbb {N}}$ based on $\{\mu _p\}_{p \in M}$ is defined via its transition operator
  2.1 $$\begin{aligned} Pf(p) := \mathbb {E}\left[ f\left( S_{k+1}\right) |S_k=p\right] = \int _{T_pM} f(\exp _p(v)) \, \mu _p(dv); \end{aligned}$$
2. The *discrete-time random walk with speed* $\alpha$ based on $\{\mu _p\}_{p \in M}$ is denoted by $\{\mathcal {S}^{(\alpha )}_k\}_{ k\in \mathbb {N}}$ and is defined via its transition operator
  2.2 $$\begin{aligned} P^{(\alpha )}f(p) := \mathbb {E}\left[ f\left( S^{(\alpha )}_{k+1}\right) \Big |S^{(\alpha )}_k=p\right] = \int _{T_pM} f(\exp _p(\alpha v)) \,\mu _p(dv); \end{aligned}$$
3. Finally, the continuous-time process $\{Z^{(\alpha )}\}_{t\ge 0}$ is defined via its generator
  2.3 $$\begin{aligned} L^{(\alpha )} f(p) := \alpha ^{-2}\left( P^{(\alpha )}f(p) - f(p)\right) . \end{aligned}$$

The process $\{S_k\}_{k\in \mathbb {N}}$ evolves as follows: whenever $S_k=p$, $S_{k+1}$ is obtained by randomly choosing $X_{k+1}$ on $T_pM$ according to the measure $\mu _p$ and following the geodesic starting at *p* in the direction $X_{k+1}$ for time 1, and analogously for the walk with speed scaled by $\alpha$.

In what follows, we want to prove weak convergence to (horizontal) Brownian motion for the continuous walk $\{Z^{(\alpha )}_t\}_{t\ge 0}$ and its horizontal lift (defined below) as $\alpha$ tends to zero. As consequence, one can obtain corresponding results for the discrete walk $\{S^{(\alpha )}_{\lfloor \alpha ^{-2}t\rfloor }\}_{t\ge 0}$ as $\alpha$ tends to zero (see Remark 3.6).

In order to proceed, we need some conditions on the distribution of increments. Because we aim at proving convergence to Brownian motion, there is a centering and variance condition. Finally, in order to prove uniform convergence of generators, it is convenient to have an additional third moment condition. More precisely, we make the following assumptions:

#### Assumption 2.2

(Centering and covariance) For every $p \in M$, the measure $\mu _p$ has zero expectation and its covariance equals the inverse metric; i.e.,

$$\begin{aligned} \int _{T_pM} v \, \mu _p(dv) = 0, \quad \int _{T_pM} v \otimes v \, \mu _p(dv) = g^{-1}(p), \end{aligned}$$

or equivalently, in any smooth coordinate system about *p*,

2.4 $$\begin{aligned} \int _{T_pM} v^i \, \mu _p(dv) = 0, \quad \int _{T_pM} v^iv^j \,\mu _p(dv) = g^{ij}(p), \quad i,j = 1, \dots , d. \end{aligned}$$

#### Assumption 2.3

(Third moment condition) The third moment of the collection of measures $\{\mu _p\}_{p\in M}$ is finite, uniformly on compacts; i.e., for all $K\subset \subset M$ compact,

$$\begin{aligned} \sup _{p\in K}\int _{T_pM} \left\Vert v\right\Vert ^3 \, \mu _p(dv) < +\infty . \end{aligned}$$

#### Remark 2.4

If $p\mapsto \mu _p$ is invariant under parallel transport as considered by Jørgensen [3] in order to mimic identically distributed increments, then if Assumptions 2.2 and 2.3 are satisfied for a single $p \in M$, then they are for all $p\in M$.

### Horizontal Lift of Geodesic Random Walks

Now that we have defined geodesic random walks on the base manifold (*M*, *g*), we can construct a new process on the total manifold $(N, \tilde{g})$ carrying a metric $\tilde{g}$ that will be specified later on. In order to define this process, we recall some terminology from Riemannian geometry.

#### Definition 2.5

A *Riemannian submersion* $\pi : (N,\tilde{g}) \rightarrow (M, g)$ is a smooth surjective map whose differential is an isomorphism

$$\begin{aligned} d\pi _u : \left( \ker d\pi _u\right) ^\perp \rightarrow T_{\pi (u)}M \end{aligned}$$

which is also an isometry. Here $\perp$ denotes the orthogonal complement with respect to the metric $\tilde{g}$ in *N*.

In the setting of Riemannian submersions, the tangent space $T_uN$ of the total manifold *N* at a point $u\in N$ splits into the *vertical* and *horizontal subspaces* as follows:

$$\begin{aligned} \mathcal {V}_u N := \ker d\pi _u, \quad \mathcal {H}_u N := (\mathcal {V}_u N)^\perp , \quad T_u N = \mathcal {H}_u N \oplus \mathcal {V}_u N. \end{aligned}$$

Their disjoint unions form two subbundles of *TN* denoted, respectively, by $\mathcal {V}N = \sqcup _{u\in N} \mathcal {V}_u N$ and $\mathcal {H}N = \sqcup _{u\in N} \mathcal {H}_uN$. This splits the metric $\tilde{g}$ on *TN* into its two factors $g_{\mathcal {V}N}$ and $g_{\mathcal {H}N}$. Denote by $\Gamma$ the space of sections. A horizontal vector field $X \in \Gamma (\mathcal {H}N)$ is $\pi$*-related* to a vector field $\overline{X} \in \Gamma (TM)$ if for any $u \in N$ it holds that

2.5 $$\begin{aligned} d\pi _u\left( X_u\right) = \overline{X}_{\pi (u)}. \end{aligned}$$

We stress that relating manifolds via a Riemannian submersion ensures that $\pi$-related tangent vectors as in (2.5) have the same norm because $d\pi _{|_{\mathcal {H}N}}$ is an isometry.

#### Remark 2.6

In several situations, the total manifold *N* comes with a natural projection map $\pi : N \rightarrow M$ defining the vertical subspaces $\mathcal {V}_u N = \ker d\pi _u$ but no specification of a metric $\tilde{g}$. One can then use any connection form $\omega$ to define the horizontal subspaces $\mathcal {H}_u N = \ker \omega _u$. Now, with the help of this choice of horizontal bundle, obtained either by the Riemannian submersion or by the specification of a connection form, one can lift any smooth curve on the base manifold to the total manifold with respect to the horizontal bundle.

We can now define the horizontal lift of a curve $\gamma : I\rightarrow M$. We denote $\gamma '(t)=\tfrac{d}{dt}(\gamma (t))$.

#### Definition 2.7

The *horizontal lift* $\tilde{\gamma }$ with respect to $\mathcal {H}N$ starting at $u_0 \in N_{\gamma (0)} = \pi ^{-1}(\{\gamma (0)\})$ of a smooth curve $\gamma : I \rightarrow M$ is the unique curve satisfying

2.6 $$\begin{aligned} \pi \circ \tilde{\gamma } = \gamma , \quad \tilde{\gamma }'(t) \in \mathcal {H}_{\tilde{\gamma }(t)}N. \end{aligned}$$

The *horizontal lift* $\tilde{v}(u, v)$ with respect to $\mathcal {H}N$ starting at *u* of a tangent vector $v \in T_{\pi (u)}M$ is given by

$$\begin{aligned} \tilde{v} := \tilde{v}(u, v) := (d\pi _u)^{-1}v \in \mathcal {H}_uN, \end{aligned}$$

which corresponds to differentiating (2.6) for any curve $\gamma$ such that $\gamma (0) = \pi (u)$ and $\gamma '(0) = v \in T_{\pi (u)}M$. One can prove that this is independent of the choice of the curve, and in particular if $\tilde{v}$ is the horizontal lift of $v \in T_p M$ starting at *u*, then for every $\gamma$ in *M* such that $\gamma (0)=p$, and $\gamma '(0)=v$, $\tilde{v}$ is initially tangent to the horizontal lift of $\gamma$ starting at *u*; $\tilde{v} = \widetilde{\gamma }'(0)$.

We recall that the horizontal lift of a geodesic under a Riemannian submersion is again a geodesic (see [10, Lemma 26.11]). It is important to notice that geodesics in *N* with initial horizontal tangent vector, are horizontal curves; i.e., the tangent vectors remain horizontal. Moreover, by the geodesic property, the tangent vector at any point of the curve is the parallel transport of the initial tangent vector.

Given a distribution of increments $\{\mu _p\}_{ p\in M}$ we define the distribution of *horizontally lifted increments* $\{\tilde{\mu }_u\}_{ u\in N}$ as follows. First draw *v* according to $\mu _{\pi (u)}$ and then lift *v* to $\tilde{v}(v,u)$. Then $\{\tilde{\mu }_u\}_{ u\in N}$ is the distribution of the lift $\tilde{v}$. It then follows that the (discrete or continuous-time) random walks based on $\{ \mu _p \}_{ p\in M }$ are horizontally lifted to the (discrete or continuous-time) random walks based on $\{ \tilde{\mu }_u \}_{ u\in N}$, and conversely, the projections of random walks based on $\{ \tilde{\mu }_u \}_{u\in N}$ are distributed as the random walks based on $\{\mu _p\}_{p\in M}$.

As a consequence, the *horizontal lift of the rescaled continuous-time random walk* $\{Z^{(\alpha )}_t\}_{ t\ge 0 }$ defined via its generator (2.3) is the process $\{\tilde{Z}^{(\alpha )}_t\}_{ t\ge 0}$ on the total manifold *N* with generator defined on smooth compactly supported functions $f:N\rightarrow \mathbb {R}$ by

2.7 $$\begin{aligned} \mathcal {L}_\alpha f(u) := \alpha ^{-2} \int _{T_{\pi (u)}M} \left( f\left( \exp _u \{\alpha \tilde{v}(v,u)\}\right) - f(u)\right) \, \mu _{\pi (u)}(dv), \end{aligned}$$

so that $\{\pi (\tilde{Z}^{(\alpha )}_t)\}_{ t\ge 0 }= \{Z^{(\alpha )}_t\}_{ t\ge 0}$ in distribution.

## Invariance Principle for Riemannian Submersions

### Main Result

Let (*M*, *g*) and $(N, \tilde{g})$ be Riemannian manifolds with Riemannian submersion $\pi : N \rightarrow M$, and let *d* be the dimension of *M*. Each vector field $X \in \Gamma (TN)$ can be uniquely decomposed into its horizontal part $X_\mathcal {H}\in \mathcal {H}$ and vertical part $X_\mathcal {V}\in \mathcal {V}$, respectively. Under this setting, we consider the Laplace-Beltrami operator $\Delta _N$ on *N* and its decomposition in horizontal and vertical parts as follows (see [2]):

#### Definition 3.1

The *horizontal Laplacian* $\Delta _\mathcal {H}$ is the generator of the pre-Dirichlet form

$$\begin{aligned} \mathcal {E}_\mathcal {H}(f, h) = -\int _N \langle ({{\,\mathrm{\textrm{grad}}\,}}f)_\mathcal {H}, ({{\,\mathrm{\textrm{grad}}\,}}h)_\mathcal {H}\rangle _{\tilde{g}} \, d\textrm{Vol}_{\tilde{g}}, \quad f, h \in C^\infty _c(N). \end{aligned}$$

Analogously, the *vertical Laplacian* $\Delta _\mathcal {V}$ is the generator of the pre-Dirichlet form

$$\begin{aligned} \mathcal {E}_\mathcal {V}(f, h) = -\int _N \langle ({{\,\mathrm{\textrm{grad}}\,}}f)_\mathcal {V}, ({{\,\mathrm{\textrm{grad}}\,}}h)_\mathcal {V}\rangle _{\tilde{g}} \, d\textrm{Vol}_{\tilde{g}}, \quad f, h \in C^\infty _c(N). \end{aligned}$$

We have the decomposition of the total Laplacian into its horizontal and vertical parts

$$\begin{aligned} \Delta _N = \Delta _\mathcal {H}+ \Delta _\mathcal {V}. \end{aligned}$$

In local orthonormal frames $\{E_i\}_{1\le i\le d}$ of $\mathcal {H}$ and $\{F_j\}_{1\le j \le l}$ of $\mathcal {V}$, the horizontal Laplacian can be rewritten as

3.1 $$\begin{aligned} \Delta _\mathcal {H}= -\sum _{i=1}^d E_i E_i^* = \sum _{i=1}^d \left( E_i^2 - \left( \nabla _{E_i} E_i\right) _\mathcal {H}\right) - \sum _{j=1}^l \left( \nabla _{F_j} F_j\right) _\mathcal {H}, \end{aligned}$$

where the adjoint is understood in $L^2(N, d\textrm{Vol}_{\tilde{g}})$.

The $F_j$’s are vertical, so they cannot be obtained as horizontal lifts. Thus, horizontally lifted geodesic random walks are not expected to produce the last term in (3.1) since their tangent vectors are horizontal. The following type of Riemannian submersion ensures that the last term indeed vanishes; $\left( \nabla _{F_j} F_j\right) _\mathcal {H}= 0$ (see [11] and [12, Proposition 4.13]).

#### Definition 3.2

The fibers $N_p:= \pi ^{-1}(\{p\})$ of a Riemannian submersion $\pi : N \rightarrow M$ are said to be *totally geodesic* if any geodesic in a fiber, seen as a submanifold of *N* with the induced metric, is also a geodesic in *N*.

Assuming that the submersion has totally geodesic fibers, the horizontal Laplacian (3.1) takes the form

3.2 $$\begin{aligned} \Delta _\mathcal {H}= \sum _{i=1}^d \left( E_i^2 - \left( \nabla _{E_i} E_i\right) _\mathcal {H}\right) . \end{aligned}$$

#### Definition 3.3

A distribution $\Lambda$ of the tangent bundle *TN* is said to be *bracket-generating* if it is generated by a finite number of Lie brackets of vector fields in $\Gamma (\Lambda )$.

Whenever the horizontal subbundle $\mathcal {H}N$ of *TN* is bracket-generating, the subellipticity of $\Delta _\mathcal {H}$ is then guaranteed by Hörmander’s theorem. Moreover, [2, Proposition 4.1.5] guarantees in that case its self-adjointness on $C_c^\infty (N)$, and its associated pre-Dirichlet form has a unique closed extension. On the other hand, as $\mathcal {V}$ is never bracket-generating, we will not consider vertically lifted geodesic random walks.

We are now ready to state the invariance principle for the horizontal lift of the rescaled continuous-time random walk for these types of Riemannian submersions. As a corollary, we obtain the associated relation between the Laplace–Beltrami operator and the horizontal Laplacian.

#### Theorem 3.4

(Invariance principle for Riemannian submersions) Let (*M*, *g*) and $(N,\tilde{g})$ be geodesically complete Riemannian manifolds equipped with a Riemannian submersion $\pi : N \rightarrow M$ with totally geodesic fibers such that the horizontal subbundle $\mathcal {H}N$ of *TN* is bracket-generating. Let $\{\mu _p\}_{p\in M}$ be a distribution of increments on *M* satisfying Assumption 2.2 and Assumption 2.3. Let $\{\tilde{Z}^{(\alpha )}_t\}_{t\ge 0}$ be the process with generator (2.7). Then as $\alpha \rightarrow 0$, this process converges to horizontal Brownian motion; i.e., the process with generator $\frac{1}{2}\Delta _{\mathcal {H}}$.

#### Corollary 3.5

Under the setting of Theorem 3.4, the following identity holds:

3.3 $$\begin{aligned} \Delta _\mathcal {H}\left( f \circ \pi \right) = (\Delta _M f) \circ \pi , \quad f \in C^\infty (M). \end{aligned}$$

#### Proof of Corollary 3.5

Consider an $\alpha$-rescaled continuous-time random walk $\{Z^{(\alpha )}_t\}_{t\ge 0}$ on *M* that satisfies Assumption 2.2 and Assumption 2.3. By Theorem 3.4 with ${{\,\mathrm{\textrm{Id}}\,}}_M: M \rightarrow M$ as submersion, this process converges to Brownian motion; i.e., the process with generator $\tfrac{1}{2}\Delta _M$. On the other hand, by Theorem 3.4, the horizontal lift of this $\alpha$-rescaled random walk, namely, the process $\{\tilde{Z}^{(\alpha )}_t\}_{t\ge 0}$ on *N*, converges to horizontal Brownian motion; i.e., the process with generator $\tfrac{1}{2}\Delta _\mathcal {H}$ on *N*. Because the projection $\pi : N \rightarrow M$ is continuous, and because the random walks $\{Z^\alpha _t\}_{t\ge 0}$ and $\{\tilde{Z}^{(\alpha )}_t\}_{t\ge 0}$ are by construction $\pi$-related, the corresponding limiting Brownian motions must be $\pi$-related as well. This proves the identity (3.3) for the case of smooth compactly supported functions, and, since (3.3) is a pointwise identity, this is enough.

#### Proof of Theorem 3.4

Let $f: N \rightarrow \mathbb {R}$ be a smooth compactly supported function. We perform a Taylor expansion of $f \circ \widetilde{\gamma _\alpha }$ around $u \in N_p$, where $\widetilde{\gamma _\alpha }$ is the horizontal lift starting at *u* of the curve $\gamma _\alpha (t) = \exp _p(\alpha tv)$, where $v \in T_pM$. There is some $0< s < 1$ such that

$$\begin{aligned} f \left( \widetilde{\gamma _\alpha }(1)\right) - f(u) = \left( \left. \frac{d}{dt}\right| _{t=0} + \frac{1}{2} \left. \frac{d^2}{dt^2}\right| _{t=0} + \frac{1}{6} \left. \frac{d^3}{dt^3}\right| _{t=s}\right) f\left( \widetilde{\gamma _\alpha }(t)\right) . \end{aligned}$$

The first time derivative is given by

$$\begin{aligned} \frac{d}{dt} f\left( \widetilde{\gamma _\alpha }(t)\right)&= df_{\widetilde{\gamma _\alpha }(t)} \circ \left( d\pi _{\widetilde{\gamma _\alpha }(t)}\right) ^{-1}(\gamma _\alpha '(t)) = \left\langle {{\,\mathrm{\textrm{grad}}\,}}f, \widetilde{\gamma _\alpha }'(t) \right\rangle _{\tilde{g}} \\  &= \left\langle \left( {{\,\mathrm{\textrm{grad}}\,}}f\right) _\mathcal {H}, \widetilde{\gamma _\alpha }'(t) \right\rangle _{\tilde{g}}, \end{aligned}$$

where we used the fact that $\widetilde{\gamma _\alpha }$ is horizontal for the last equality.

To obtain the second time derivative, we use the Levi–Civita connection $\nabla$ on *N* and use the fact that $\widetilde{\gamma _\alpha }$ is a geodesic being the horizontal lift of a geodesic under a Riemannian submersion;

$$\begin{aligned} \frac{d^2}{dt^2} f \left( \widetilde{\gamma _\alpha }(t)\right)&= \left\langle \nabla _{\widetilde{\gamma _\alpha }'(t)} ({{\,\mathrm{\textrm{grad}}\,}}f)_\mathcal {H}, \widetilde{\gamma _\alpha }'(t)\right\rangle _{\tilde{g}} + \left\langle ({{\,\mathrm{\textrm{grad}}\,}}f)_\mathcal {H}, \nabla _{\widetilde{\gamma _\alpha }'(t)} \widetilde{\gamma _\alpha }'(t)\right\rangle _{\tilde{g}} \\&= \left\langle \nabla _{\widetilde{\gamma _\alpha }'(t)} ({{\,\mathrm{\textrm{grad}}\,}}f)_\mathcal {H}, \widetilde{\gamma _\alpha }'(t)\right\rangle _{\tilde{g}}. \end{aligned}$$

In particular, at time $t = 0$, consider the orthonormal basis $\{E_i\}_{1\le i\le d}$ of $\mathcal {H}_uN \subset T_uN$ defined as the horizontal lift of an orthonormal basis $\{\overline{E}_i\}_{1\le i\le d}$ of $T_pM$. Write $v = v^i\overline{E}_i \in T_pM$. By linearity of the horizontal lift, we get $\widetilde{\gamma _\alpha }'(0) = \alpha v^iE_i$, and thus

$$\begin{aligned} \left. \frac{d^2}{dt^2}\right| _{t=0} f\left( \widetilde{\gamma _\alpha }(t)\right) = \alpha ^2 v^iv^j \left\langle \nabla _{E_i} ({{\,\mathrm{\textrm{grad}}\,}}f)_\mathcal {H}, E_j\right\rangle _{\tilde{g}} = \alpha ^2 v^iv^j \left( E_iE_j - \left( \nabla _{E_i} E_j\right) _\mathcal {H}\right) f. \end{aligned}$$

By Assumption 2.2 on the first and second moments, we deduce that

$$\begin{aligned} \alpha ^{-2}\int _{T_pM} \left. \frac{d}{dt}\right| _{t=0} f\left( \widetilde{\gamma _\alpha }(t)\right) \, \mu _p(dv) = \alpha ^{-1} df_u \circ \left( d\pi _u\right) ^{-1} \left( \int _{T_pM}v \, \mu _p(dv)\right) = 0, \end{aligned}$$

and

$$\begin{aligned} \alpha ^{-2} \int _{T_pM} \left. \frac{d^2}{dt^2}\right| _{t=0} f \left( \widetilde{\gamma _\alpha }(t)\right) \, \mu _p(dv)&= \left( \int _{T_pM} v^iv^j \mu _p(dv)\right) \left( E_iE_j - \left( \nabla _{E_i}E_j\right) _\mathcal {H}\right) f \\&=\sum _{i=1}^d \left( E_i^2 - \left( \nabla _{E_i}E_i\right) _\mathcal {H}\right) f. \end{aligned}$$

The last term is the horizontal Laplacian (3.2) for a submersion with totally geodesic fibers.

For the third time derivative, first define the horizontal Hessian

$$\begin{aligned} {{\,\mathrm{\textrm{Hess}}\,}}_\mathcal {H}f(Y, Z) = \left\langle \nabla _Y \left( {{\,\mathrm{\textrm{grad}}\,}}f\right) _\mathcal {H}, Z\right\rangle _{\tilde{g}}, \quad Y, Z \in \Gamma (TN), \end{aligned}$$

which is a symmetric covariant tensor of order 2. Its covariant derivative is thus the tensor given by

$$\begin{aligned} \nabla {{\,\mathrm{\textrm{Hess}}\,}}_\mathcal {H}f(X, Y, Z) = X\left( {{\,\mathrm{\textrm{Hess}}\,}}_\mathcal {H}f(Y, Z)\right) - {{\,\mathrm{\textrm{Hess}}\,}}_\mathcal {H}f(\nabla _X Y, Z) - {{\,\mathrm{\textrm{Hess}}\,}}_\mathcal {H}f(Y, \nabla _X Z). \end{aligned}$$

Note that, again since $\widetilde{\gamma _\alpha }$ is a geodesic,

$$\begin{aligned}&\frac{d^3}{dt^3} f(\widetilde{\gamma _\alpha }(t)) = \nabla _{\widetilde{\gamma _\alpha }'(t)} \left\langle \nabla _{\widetilde{\gamma _\alpha }'(t)}\left( {{\,\mathrm{\textrm{grad}}\,}}f\right) _\mathcal {H}, \widetilde{\gamma _\alpha }'(t)\right\rangle _{\tilde{g}} \\&\quad = \nabla _{\widetilde{\gamma _\alpha }'(t)} \left\langle \nabla _{\widetilde{\gamma _\alpha }'(t)}\left( {{\,\mathrm{\textrm{grad}}\,}}f\right) _\mathcal {H}, \widetilde{\gamma _\alpha }'(t)\right\rangle _{\tilde{g}} - 2\left\langle \nabla _{\nabla _{\widetilde{\gamma _\alpha }'(t)} \widetilde{\gamma _\alpha }'(t)}\left( {{\,\mathrm{\textrm{grad}}\,}}f\right) _\mathcal {H}, \widetilde{\gamma _\alpha }'(t) \right\rangle _{\tilde{g}} \\&\quad = \nabla {{\,\mathrm{\textrm{Hess}}\,}}_\mathcal {H}f\left( \widetilde{\gamma _\alpha }'(t), \widetilde{\gamma _\alpha }'(t), \widetilde{\gamma _\alpha }'(t)\right) . \end{aligned}$$

Locally, $(\nabla {{\,\mathrm{\textrm{Hess}}\,}}_\mathcal {H}f)_u: T^3_uN \rightarrow \mathbb {R}$ is a bounded operator being linear on a finite dimensional vector space with operator norm given by

$$\begin{aligned} C(u) = \max _{(\eta , \xi , \zeta ) \in T^3_uN \, : \,\left\Vert \eta \right\Vert _{\widetilde{g}} = \left\Vert \xi \right\Vert _{\widetilde{g}} = \left\Vert \zeta \right\Vert _{\widetilde{g}} = 1} \left| \left( \nabla {{\,\mathrm{\textrm{Hess}}\,}}_\mathcal {H}f\right) _u(\eta , \xi , \zeta )\right| . \end{aligned}$$

This constant *C*(*u*) can be uniformly bounded since $|\nabla {{\,\mathrm{\textrm{Hess}}\,}}_\mathcal {H}f|: N \times T^3N \rightarrow \mathbb {R}$ is a continuous map on the compact set $\{(q,(\eta , \xi , \zeta )) \,: \, q \in {{\,\mathrm{\textrm{Supp}}\,}}f, (\eta , \xi , \zeta ) \in T^3_qN, \left\Vert \eta \right\Vert _{\widetilde{g}} = \left\Vert \xi \right\Vert _{\widetilde{g}} = \left\Vert \zeta \right\Vert _{\widetilde{g}} = 1\}$, and hence attains a maximum $C > 0$. Since $\left\Vert \widetilde{\gamma _\alpha }'(t)\right\Vert _{\tilde{g}} = \left\Vert \widetilde{\gamma _\alpha }'(0)\right\Vert _{\tilde{g}} = \alpha \left\Vert v\right\Vert _{g}$, we are able to conclude by Assumption 2.3 on the third moment;

$$\begin{aligned}&\alpha ^{-2}\int _{T_pM} \left. \frac{d^3}{dt^3}\right| _{t=s} f\left( \widetilde{\gamma _\alpha }(t)\right) \mu _p(dv) \\  &\quad = \alpha ^{-2} \int _{T_pM} \nabla {{\,\mathrm{\textrm{Hess}}\,}}_\mathcal {H}f\left( \widetilde{\gamma _\alpha }'(s), \widetilde{\gamma _\alpha }'(s), \widetilde{\gamma _\alpha }'(s)\right) \mu _p(dv) \\&\quad \le \alpha C\sup _{q \in \pi \left( {{\,\mathrm{\textrm{Supp}}\,}}f\right) } \int _{T_qM} \left\Vert v\right\Vert _g^3 \, \mu _q(dv), \end{aligned}$$

which goes to 0 independently of *u* as $\alpha \rightarrow 0$.

#### Remark 3.6

The transition operator of the discrete-time random walk $\{\mathcal {S}^{(\alpha )}_k\}_{ k\in \mathbb {N}}$ is related to the generator of the continuous-time process $\{Z_t^{(\alpha )}\}_{t \ge 0}$ via (2.3):

$$\begin{aligned} P^{(\alpha )} = \left( \textrm{Id} + \alpha ^2 L^{(\alpha )}\right) . \end{aligned}$$

The strong convergence of the generator $L^{(\alpha )}$ toward $\mathcal {L}$ on its core $C_c^\infty (M)$ then implies that $(P^{(\alpha )})^{\lfloor \alpha ^{-2}t\rfloor }$ converges to the semigroup $e^{t\mathcal {L}}$ by the Trotter-Kurtz theorem. Analogously, the same holds for the horizontally lifted discrete-time random walk.

#### Remark 3.7

We obtained Corollary 3.5 as a consequence of the invariance principle, which contrasts with the classical approach. For the sake of completeness, we now briefly outline the latter. Essentially, the proof reduces to showing that the Levi-Civita connection $\nabla$ on *N* is $\pi$-related to the Levi–Civita connection $\overline{\nabla }$ on *M* (see [11, Lemma 1]). This follows from the fact that both the inner products for the specific metrics and the Lie brackets preserve $\pi$-relations;

$$\begin{aligned} \left\langle X, W \right\rangle _{\tilde{g}} = \left\langle \overline{X}, \overline{W}\right\rangle _g \circ \pi , \quad d\pi \left( \left[ Y, Z\right] \right) = \left[ \overline{Y}, \overline{Z}\right] \circ \pi \end{aligned}$$

for $\pi$-related vector fields $W, X, Y, Z \in \Gamma (\mathcal {H}N)$ to $\overline{W}, \overline{X}, \overline{Y}, \overline{Z} \in \Gamma (TM)$, and hence

$$\begin{aligned} \left\langle X, [Y, Z] \right\rangle _{\tilde{g}} = \left\langle \overline{X}, [\overline{Y}, \overline{Z}] \right\rangle _g \circ \pi . \end{aligned}$$

It remains to express the Levi-Civita connection $\nabla$ on *N* via Koszul’s formula for any triple $X, Y, Z \in \Gamma (TN)$;

$$\begin{aligned} 2\left\langle \nabla _X Y,Z\right\rangle _{\tilde{g}}&= X\langle Y,Z\rangle _{\tilde{g}} + Y\langle X, Z\rangle _{\tilde{g}} - Z\langle X,Y\rangle _{\tilde{g}} \\&\quad -\left\langle X, [Y,Z]\right\rangle _{\tilde{g}} -\left\langle Y, [X, Z]\right\rangle _{\tilde{g}} + \left\langle Z, [X, Y]\right\rangle _{\tilde{g}} . \end{aligned}$$

### Important Examples

Let us go through some important examples from [2, Sections 4.1, 4.4] where the restrictions on the Riemannian submersion, namely, that the fibers are totally geodesic and that the horizontal distribution is bracket-generating, are verified.

1. The manifold (*M*, *g*) itself, with ${{\,\mathrm{\textrm{Id}}\,}}_M: M \rightarrow M$ as submersion. The horizontal distribution is the whole tangent space. Theorem 3.4 gives then a proof of the invariance principle for geodesic random walks on Riemannian manifolds.
2. The tangent bundle $\pi _{TM}: TM \rightarrow M$ equipped with the Sasaki metric [13] defined in terms of coordinates $(\{x_i\}_{1\le i\le d}, \{y_j\}_{1\le j\le d})$ about (*p*, *v*) in *TM* by
  $$\begin{aligned} ds^2 = g_{ij}dx^i dx^j + g_{ij}Dy^i Dy^j, \end{aligned}$$
  where *D* denotes the covariant differential with respect to $\overline{\nabla }$ on *M*; $Dy^k = dy^k + \overline{\Gamma }^k_{ij}y^i dx^j$.
3. The orthonormal frame bundle $\pi _{O(M)}: O(M) \rightarrow M$ which plays a central role in defining stochastic processes on manifolds by constructing them from their Euclidean counterparts. This motivated our study of horizontal random walks.

#### Definition 3.8

An *orthonormal frame* *u* at *p* is an ordered choice of orthonormal basis $\{ue_i\}_{1\le i\le d}$ of $T_pM$, where $\{e_i\}_{1\le i\le d}$ is the canonical basis of $\mathbb {R}^d$. The set of all orthonormal frames at *p* is denoted $O_p$ and their disjoint union $O(M):= \sqcup _{p \in M} O_p$ is referred to as the *orthonormal frame bundle*.

The orthonormal frame bundle *O*(*M*) is a manifold of dimension $d(d+1)/2$ that comes with a natural submersion $\pi _{O(M)}: O(M) \rightarrow M$ sending any orthonormal frame $u \in O_p$ to the basepoint *p*. If $(U, \{x^i\}_{1\le i\le d})$ is a local chart in *M* about *p*, we can express the orthonormal basis of $T_pM$ as $ue_i = e_i^j \frac{\partial }{\partial x^j}$, where $e^j_i = (ue_i)^j$, and this gives a local chart $(\pi ^{-1}(U), (\{x^k\}_{1\le k\le d}, \{e_i^j\}_{1\le i<j\le d}))$ in *O*(*M*) about *u*. It remains to define a splitting of *TO*(*M*), for instance, by specifying a notion of horizontality.

#### Definition 3.9

A smooth curve $u: I \rightarrow O(M)$ is *horizontal* if for any $e\in \mathbb {R}^d$ the tangent vector field $u(t)e \in T_{\pi (u(t))}M$ is itself parallel with respect to the Levi-Civita connection $\overline{\nabla }$ on *M* along the curve $\pi \circ u: I \rightarrow M$.

This notion of horizontality induces the splitting $TO(M) = \mathcal {H}O(M) \oplus \mathcal {V}O(M)$ and allows us to lift smooth curves horizontally. Given a smooth $\gamma : I \rightarrow M$ and its horizontal lift $\tilde{\gamma }$ starting at *u*, we recover the parallel transport of tangent vectors along $\gamma$ given by

$$\begin{aligned} \tau _{\gamma ; t_1t_2} : T_{\gamma (t_1)} \rightarrow T_{\gamma (t_2)} : v \mapsto \tilde{\gamma }(t_2)\left( \tilde{\gamma }(t_1)\right) ^{-1}v. \end{aligned}$$

Note that for each frame *u*, the collection $\{ue_i\}_{i=1, \dots , d}$ is a basis of $T_{\pi (u)}M$, and thus one can horizontally lift each of those tangent vectors in order to obtain a new collection of horizontal vectors. In this way, one creates the globally defined canonical horizontal vector fields.

#### Definition 3.10

Let *u* be an orthonormal frame at *p*. The *canonical horizontal vector fields*

3.4 $$\begin{aligned} H_i(u) := \widetilde{ue_i}, \quad i = 1,\dots ,d, \end{aligned}$$

are the horizontal lifts with respect to $\mathcal {H}O(M)$ of the tangent vectors $ue_i \in T_pM$ starting at *u*.

The global canonical horizontal vector fields $H_i \in \Gamma (\mathcal {H}O(M))$ allow us to obtain a horizontal Laplacian for the orthonormal frame bundle as a sum of squares;

#### Definition 3.11

The horizontal Laplacian of *O*(*M*) is given by

$$\begin{aligned} \Delta _\mathcal {H}:= \sum _{i=1}^d H_i^2. \end{aligned}$$

#### Remark 3.12

Note that $\sum _{i=1}^d H_i^2$ is in *Hörmander’s form*, and differs from (3.2) in the general case of submersions with totally geodesic fibers; this is due to the fact that *O*(*M*) is a parallelizable manifold. While Nash’s embedding theorem allows one to write the Laplace–Beltrami operator of *M* as a sum of squares of orthogonal projections (see for instance [1, Theorem 3.1.4]), this comes at cost of extra terms coming from the dimension of the isometric embedding.

This horizontal Laplacian and the Laplace–Beltrami operator satisfy the following relation, and this is a starting point in stochastic calculus on manifolds based on the rolling-without-slipping construction of Brownian motion as described in Hsu’s monograph [1]:

#### Proposition 3.13

The following identity holds:

3.5 $$\begin{aligned} \Delta _\mathcal {H}\left( f \circ \pi _{O(M)}\right) = \Delta _M f \circ \pi _{O(M)}, \quad f \in C^\infty (M). \end{aligned}$$

One can show that Definition 3.11 coincides with (3.1) once a specific metric is prescribed, namely, the Sasaki-Mok metric (specified below in more detail). This means the invariance principle for horizontal geodesic random walks on *O*(*M*) are a consequence of Theorem 3.4. However, it turns out that for *O*(*M*) a more direct proof of this invariance principle is available without relying on this metric and the fact that the fibers are totally geodesic. We will detail this proof as follows:

In particular, Proposition 3.13 is a corollary of that invariance principle just as before. Given $f \in C^\infty (O(M))$ and $\widetilde{\gamma _\alpha }(t)$ the horizontal lift starting at the frame $u \in \pi _{O(M)}^{-1}(\{p\})$ of the geodesic $\gamma$ with $\gamma '(0) = v \in T_pM$, then by definition of horizontal lift

$$\begin{aligned} \gamma _\alpha '(t) = \tau _{\gamma _\alpha ; 0t} \left( \alpha v\right) = \widetilde{\gamma _\alpha }(t)\left( \widetilde{\gamma _\alpha }(0)\right) ^{-1} (\alpha v) = \alpha \widetilde{\gamma _\alpha }(t) u^{-1}v = \alpha \left( u^{-1} v\right) ^i \widetilde{\gamma _\alpha }(t) e_i. \end{aligned}$$

Since *u* is an orthonormal frame, $\{ue_i\}_{1\le i\le d}$ is an orthonormal basis of $T_pM$, and we get

$$\begin{aligned} v^i = \langle v, ue_i \rangle _g = \langle u^{-1}v, e_i\rangle _{\mathbb {R}^d} = \left( u^{-1} v\right) ^i. \end{aligned}$$

Therefore,

$$\begin{aligned} \frac{d}{dt} f \left( \widetilde{\gamma _\alpha }(t)\right) = df_{\widetilde{\gamma _\alpha }(t)} \circ \widetilde{\gamma _\alpha }'(t) = \alpha v^i H_i f(\widetilde{\gamma _\alpha }(t)), \end{aligned}$$

and likewise,

$$\begin{aligned}&\frac{d^2}{dt^2} f\left( \widetilde{\gamma _\alpha }(t)\right) = \alpha ^2 v^jv^i H_j H_i f(\widetilde{\gamma _\alpha }(t)), \quad \\&\frac{d^3}{dt^3} f\left( \widetilde{\gamma _\alpha }(t)\right) = \alpha ^3\ v^kv^jv^i H_k H_j H_i f(\widetilde{\gamma _\alpha }(t)). \end{aligned}$$

By the Assumption 2.2, the first moment vanishes, the second moment is given by

$$\begin{aligned} \alpha ^{-2} \int _{T_pM} \left. \frac{d^2}{dt^2}\right| _{t=0} f \left( \widetilde{\gamma _\alpha }(t)\right) \,\mu _p(dv)&= \left( \int _{T_pM} v^jv^i \,\mu _p(dv)\right) H_j H_i f(u) \\&= \sum _{i=1}^d H_i^2 f(u), \end{aligned}$$

and for the third moment we argue as follows: $\pi _{O(M)}\left( {{\,\mathrm{\textrm{Supp}}\,}}f\right)$ is compact by continuity of the projection, and thanks to Assumption 2.3 we estimate

$$\begin{aligned}&\alpha ^{-2}\int _{T_pM} \left. \frac{d^3}{dt^3}\right| _{t=s} f \left( \widetilde{\gamma _\alpha }(t)\right) \,\mu _p(dv)\\&\qquad = \alpha \int _{T_pM} v^kv^jv^i H_k H_j H_i f\left( {\widetilde{\gamma }_\alpha }(s)\right) \,\mu _p(dv) \\&\qquad \le \alpha \sup _{q\in \pi _{O(M)}\left( {{\,\mathrm{\textrm{Supp}}\,}}f\right) } \int _{T_qM} \left\Vert v\right\Vert ^3 \,\mu _q(dv) \sum _{i,j,k=1}^d\left\Vert H_k H_j H_i f\right\Vert _\infty , \end{aligned}$$

which goes to 0 independently of the frame *u* as $\alpha \rightarrow 0$.

For the sake of completeness, we now describe this metric which was introduced by Mok [14] (see also Sasaki [13] and [15]). One starts by finding a coordinate expression for the canonical horizontal vector fields. Consider a horizontal lift $\tilde{\gamma }: I \rightarrow O(M)$ that starts at *u* with $\gamma '(0) = ue_i$. By definition of horizontal lift with respect to $\mathcal {H}O(M)$,

$$\begin{aligned} H_i(u) = \widetilde{ue_i} = \tilde{\gamma }'(0) = \dot{\gamma }^j(0)\frac{\partial }{\partial x^j} + \left( \left( \tilde{\gamma }e_l\right) ^m\right) '(0)\frac{\partial }{\partial e_l^m}, \end{aligned}$$

and since the tangent vectors $\tilde{\gamma }(t)e_l$ are parallel with respect to $\overline{\nabla }$ on *M* along the curve $\pi \circ \tilde{\gamma }: I \rightarrow M$ whose initial tangent vector is $ue_i$, the geodesic equation yields

$$\begin{aligned} \left( \left( \tilde{\gamma }e_l\right) ^m\right) '(0) = -e_i^je_l^k\overline{\Gamma }^m_{jk}, \quad 1 \le l < m \le d, \end{aligned}$$

where $\{\overline{\Gamma }^k_{ij}\}_{1\le i,j,k\le d}$ denote the Christoffel symbols of $\overline{\nabla }$. The horizontal and vertical subbundles of *TO*(*M*) are thus, respectively, spanned by (see [1, Proposition 2.1.3])

3.6 $$\begin{aligned} H_i(u) = e_i^j \left( \frac{\partial }{\partial x^j} - e_l^k\overline{\Gamma }_{jk}^m\frac{\partial }{\partial e_l^m}\right) , \quad i = 1, \dots , d, \end{aligned}$$

and

3.7 $$\begin{aligned} \quad V^k_j(u) := e_j^l\frac{\partial }{\partial e_k^l}, \quad 1 \le j < k \le d. \end{aligned}$$

#### Definition 3.14

The *canonical* 1*-form* $\theta$ and the *connection form* $\omega$ on *O*(*M*) associated with $\overline{\nabla }$ on *M* are the dual forms to the vector fields (3.6) and (3.7) given by

$$\begin{aligned} \theta ^k(u) := e_l^k dx^l, \quad \omega ^j_i(u) := e_k^j\left( \overline{\Gamma }^k_{lm} e_i^l dx^m + de_i^k\right) . \end{aligned}$$

The *Sasaki-Mok metric* $\tilde{g}$ is defined pointwise by

$$\begin{aligned} \langle \eta , \xi \rangle _{\tilde{g}} := \left\langle d\pi _{O(M),u} (\eta ) , d\pi _{O(M),u}(\xi ) \right\rangle _g + \left\langle \omega _u(\eta ), \omega _u(\xi )\right\rangle , \end{aligned}$$

where $\langle \cdot , \cdot \rangle$ denotes an *O*(*d*)-invariant inner product on $\mathfrak {o}(d)$.

With this metric and connection form, one can prove that $\pi _{O(M)}: O(M) \rightarrow M$ is a totally geodesic submersion. As consequence, the invariance principle for lifted geodesic random walk with limiting generator $\tfrac{1}{2}\Delta _\mathcal {H}$ follows from Theorem 3.4 and therefore also the equality between Definition 3.11 and (3.1).

4. A general class of spaces on which such invariance principle holds are the principal bundles $\pi _P: P \rightarrow M$ with fiber Lie group *G*. Given a *G*-compatible connection form $\omega$ and a *G*-invariant metric *b* on *G*, there is a unique metric $\tilde{g} = \pi ^*g + b\omega$ on *P* that makes $\pi _P$ into a Riemannian submersion with totally geodesic fibers such that the horizontal distribution $\mathcal {H}$ of $\omega$ is the orthogonal complement of the vertical distribution [16, Theorem 3.5]. Whenever the horizontal distribution $\mathcal {H}$ is bracket-generating, the subellipticity of $\Delta _\mathcal {H}$ is guaranteed and there is a unique closed extension of its associated pre-Dirichlet form. The previous examples fall under this category.

## Data Availability

No datasets were generated or analyzed during the current study.
